# Boric Acid and the Vermiform Appendix

**Published:** 1906-06

**Authors:** 


### Boric Acid and the Vermiform Appendix.

[*Editorial New York Medical Journal, April 7, 1906.*]

WE think is is generally admitted that appendicular inflammation is on the increase. Certainly the diagnosis of the condition is. The increase cannot be accounted for by any developmental or anatomical change in the organ, and there has been no material change in the staple articles of our diet. But it is worthy of note that during the last few years, *pari passu* with the actual increase of the affection, we have introduced new methods of preserving our food, methods affecting all classes of society quite as indiscriminately as the disease itself does. Statistics are said to prove that appendicular inflammation is more prevalent during summer and in hot climates. Such would be either the time or place when and where an extra dose of some preservative drug would be mixed with food to arrest decomposition. That brings forward the question, What is the most common preservative employed? The answer undoubtedly is, boric acid, for salicylic acid and formic aldehyde are much less in vogue. Boric acid is reputed to be the antiseptic constituent of a common food preservative. Moreover, the drug is more or less freely used to preserve milk, cream, butter, meat, fish, and even soups. In fact, in warm weather it would be difficult for any individual to escape ingestion of the drug.

Let us now consider what may be the effects of boric acid on the alimentary tract when given in small medicinal doses for, sometimes, a short continuous period. It has been recorded that fourteen grains daily will quickly produce pain in the epigastrium and gas in the stomach and intestines, together with colic and diarrhea, or in other words, act as an irritant of the gastro-intestinal tract. Other consequences which have been noted from its use are albuminuria and an erythematous or vesicular eruption, together with the appearance of vesicles in the mouth and on the fauces. Possibly this vesiculation may extend to other parts of the digestive tract. There is no getting away from the fact that boric acid possesses the power of irritating the alimentary canal. This has led us to wonder whether boric acid can directly or indirectly have any part in the production of appendicular inflammation. The various forms of the disease—the simple, or catarrhal, stage with plastic peritonitis, the ulcerative condition with local abscess, or the gangrenous and perforated appendix, leading to diffuse septic peritonitis—are but degrees of an affection which lowers the vitality of the tissues and renders them more or less vulnerable to the onslaught of the *Bacillus coli communis.* Under normal intestinal conditions this organism does not seem to be harmful, but, given a suitable change in its surroundings, such as an inflamed mucous membrane, or possibly under stimulation by some change in the intestinal secretions due to antiseptics, it takes on an agressive, penetrative action.

We believe we are correct in saying that the addition of antiseptics to sewage has in a measure been condemned because the ultimate sterilisation by Nature's own agents, the contained germs, is thereby arrested. Perhaps a similar upsetting of normal conditions may be caused in the human cesspool by altering the conditions under which the *Bacillus coli communis* was intended to work. We know but little of the physiological functions of the appendix, although its anatomy is minutely worked out. Foreign bodies are not so frequently reported as being found nowadays in the appendix. The fecal concretion has taken first place. Is there any reason for this mass forming? The valve of Gerlach is presumably intended to guard the entrance of the appendix, and one would naturally deduce that its *raison d'être* was to prevent gravitation of fecal material or foreign bodies from above. But if the cecum becomes distended by gas, such as boric acid would produce, the valve would theoretically become incompetent and be drawn open. With the law of gravity coming into force the superjacent feces would be more likely to invade it. Now, if peristalsis proves equal to their subsequent ejection, all may be well. But if not, there remains in the appendix material for developing into the fecal concretion.

Should the condition be simply one of catarrh, one or another result may happen—either the mucus is extruded or the cavity of the appendix becomes distended by it. Now, boric acid does set up an inflammatory condition of the intestines, and it is quite likely that it may cause the condition in the cecum and appendix. One point that has appealed to us as a very weak one in the suggestion is that young children, who take a large proportion of their diet in the shape of milk food are comparatively free from appendicitis. One would not expect this, since boric acid is so frequently added to milk, and consequently they should be more exposed to its action. But perhaps their apparent immunity may be due to intuition, in that a child, when its stomach gets out of order, immediately refuses its food, and does by instinct what an older person has to be told to, namely, give the bowel rest by starvation, or possibly to the saving effects of that sovereign remedy, a timely dose of castor oil.

